# Application of deep learning convolutional neural networks to identify gastric squamous cell carcinoma in mice

**DOI:** 10.3389/fmed.2025.1587417

**Published:** 2025-05-13

**Authors:** Yuke Ren, Shuangxing Li, Di Zhang, Yongtian Zhao, Yanwei Yang, Guitao Huo, Xiaobing Zhou, Xingchao Geng, Zhi Lin, Zhe Qu

**Affiliations:** ^1^National Institutes for Food and Drug Control, Chinese Academy of Medical Sciences & Peking Union Medical College, Beijing, China; ^2^National Center for Safety Evaluation of Drugs, State Key Laboratory of Drug Regulatory Science, National Institutes for Food and Drug Control, Beijing, China; ^3^Indica Labs, Inc., Albuquerque, NM, United States; ^4^Institute for Biological Product Control, National Institutes for Food and Drug Control, Beijing, China

**Keywords:** non-clinical safety evaluation of drugs, toxicological pathology, artificial intelligence, deep learning, gastric squamous cell carcinoma

## Abstract

**Objective:**

In non-clinical safety evaluation of drugs, pathological result is one of the gold standards for determining toxic effects. However, pathological diagnosis might be challenging and affected by pathologist expertise. In carcinogenicity studies, drug-induced squamous cell carcinoma (SCC) of the mouse stomach represents a diagnostic challenge for toxicopathologists. This study aims to establish a detection model for mouse gastric squamous cell carcinoma (GSCC) using deep learning algorithms, to improve the accuracy and consistency of pathological diagnoses.

**Methods:**

A total of 93 cases of drug-induced mouse GSCC and 56 cases of normal mouse stomach tissue from carcinogenicity studies were collected. After scanning into digital slides, semi-automated data annotation was performed. All images underwent preprocessing, including tissue extraction, artifact removal, and exclusion of normal epithelial regions. The images were then randomly divided into training, validation, and test sets in an 8:1:1 ratio. Five different convolutional neural networks (CNNs)-FCN, LR-ASPP, DeepLabv3+, U-Net, and DenseNet were applied to identify GSCC and non-GSCC regions. Tumor prediction images (algorithm results shown as overlays) derived from the slide images were compared, and the performance of the constructed models was evaluated using Precision, Recall, and F1-score.

**Results:**

The Precision, Recall, and F1-scores of DenseNet, U-Net, and DeepLabv3 + algorithms were all above 90%. Specifically, the DenseNet model achieved an overall Precision of 0.9044, Recall of 0.9291, and F1-score of 0.9157 in the test set. Compared to the other algorithms, DenseNet exhibited the highest F1-score and Recall, demonstrating superior generalization ability.

**Conclusion:**

The DenseNet algorithm model developed in this study shown promising application potential for assisting in the diagnosis of mouse GSCC. As artificial intelligence (AI) technology continues to advance in non-clinical safety evaluation of drugs, CNN-based toxicological pathology detection models will become essential tools to assist pathologists in precise diagnosis and consistency evaluation.

## 1 Introduction

Toxicologic pathology evaluation is a critical component of drug safety evaluation and serves as an essential basis for determining organ-specific alteration caused by drugs. The rapid development of digital whole-slide imaging (WSI) technology and AI technology in 2016–2017 has significantly advanced the field of pathology. Currently, several AI models have been established in the field of toxicological pathology abroad to address related tasks in toxicology (1), such as hepatocyte hypertrophy (2), retinal toxicity evaluation (3), and progressive cardiomyopathy (PCM) scoring (4), among others. As a result, AI algorithm models can provide decision support to pathologists in non-clinical studies.

Mice are commonly used rodents in carcinogenicity research, and the inter-group differences in the incidence of malignant tumors are one of the key indicators for evaluating the carcinogenic potential of drugs (5, 6). Most rodent species possess a complex gastric structure, which includes a cornified (nonglandular) squamous epithelial region known as the “forestomach,” and this is a specific feature of rodent anatomy (7). The squamous epithelium in the mouse forestomach can develop squamous cell carcinoma (8). The occurrence of GSCC in mice may be associated with the administration of certain compounds, such as N-methyl-N-nitro-N-nitrosoguanidine (MNNG) combined with a high-salt diet (9), and GSCC was identified through histopathological examination in this mouse model. In recent years, there has been extensive research on the application of deep learning in human gastric cancer cases, such as U-Net-based gastric cancer gastroscopy image segmentation (10), typical AlexNet network-based hematoxylin–eosin (H&E)-stained sections image information extraction for gastric cancer recognition (11), and machine learning classification algorithms using dynamic contrast-enhanced magnetic resonance imaging (DCE-MRI) combined with immunohistochemistry (IHC), which can accurately predict the expression of CD3^+^, CD4^+^, and CD8^+^ tumor-infiltrating lymphocytes in gastric cancer (12). Algorithms based on Convolutional Neural Networks (CNN), such as FCN, LR-ASPP, DeepLabv3+, and DenseNet, have been used for detecting and classifying lesions in clinical images (WSI tissue images with different types of histological stains, such as H&E, IHC, Masson and so on) of the esophagus and larynx (13, 14), showing results nearly identical to those of pathologist diagnoses. Although there have been no reports on the use of AI-assisted diagnostic tools in gastric neoplasm detection and classification in the toxicological pathology field so far, applying CNN algorithms in non-clinical drug safety evaluation for gastric cancer auxiliary diagnosis holds the potential to reduce misdiagnosis and underdiagnosis caused by either pathologist limited experience or labor-intensive under time constraints extensive histopathologic examination. This approach might improve the consistency and accuracy for this specific histologic finding.

This study compares different deep learning algorithms based on different CNN architectures, constructs an auxiliary diagnostic model for mouse GSCC, and analyzes the performance and applicability of the model, aiming to support pathological evaluation in carcinogenicity studies and provide support for the application of AI technology in the field of drug safety evaluation.

## 2 Materials and methods

### 2.1 Data source

The mouse gastric tissue specimens used in this study were obtained from a mouse carcinogenicity study (N2018066) conducted at the National Center for Safety Evaluation of Drugs, National Institutes for Food and Drug Control, China. In this carcinogenicity experiment, 2-week-old C3H/HeN suckling mice were administered Aristolochic acid I and observed for 9 months after a single dose. Following this period, necropsy was performed, and organ tissues were processed into hematoxylin–eosin (HE)-stained tissue sections and examined for histopathological analysis. Among the stomach tissues, a total of 93 cases were diagnosed as GSCC, and 56 cases were diagnosed as normal gastric tissue. The tissue sections of both GSCC and normal tissue were independently diagnosed by two board-certified veterinary pathologists, and the results showed diagnostic consistency/consensus. This animal study was approved by the Institutional Animal Care and Use Committee (IACUC) of NCSED (Approval No. IACUC-2018-K013).

### 2.2 Diagnostic criteria for gastric squamous cell carcinoma

The diagnostic criteria for GSCC was based on the *International Harmonization of Nomenclature and Diagnostic Criteria for Lesions in Rats and Mice* (15, 16). The criteria include: (1) Exophytic and/or endophytic growth. (2) Individual tumor cells or small clusters of tumor cells breaching the basement membrane. (3) Loss of cellular differentiation with evidence of anaplasia. (4) Potential invasion into the submucosa, muscularis propria, and serosa. (5) Well-differentiated type: Morphology resembling normal squamous epithelium with irregular papillary structures, often showing central hyperkeratosis (keratin pearls). Invasive areas may contain numerous polygonal and pleomorphic cells with varying degrees of keratinization. (6) Poorly differentiated type (anaplastic type): Solid sheets or trabecular arrangements of spindle cells, leading to varying degrees of desmoplastic features. And the difficulty in identifying keratinization. (7) The exhibition of varying sizes (typically larger than normal cells) and shapes by cells. The presence of hyperchromatic, enlarged nuclei with prominent nucleoli. (8) Increased mitotic figures. (9) Potential metastasis to the abdominal cavity, regional lymph nodes, or lungs. All tissue samples excluded benign proliferative lesions such as gastric squamous epithelial hyperplasia and gastric papilloma.

### 2.3 Study design

**Figure 1:**
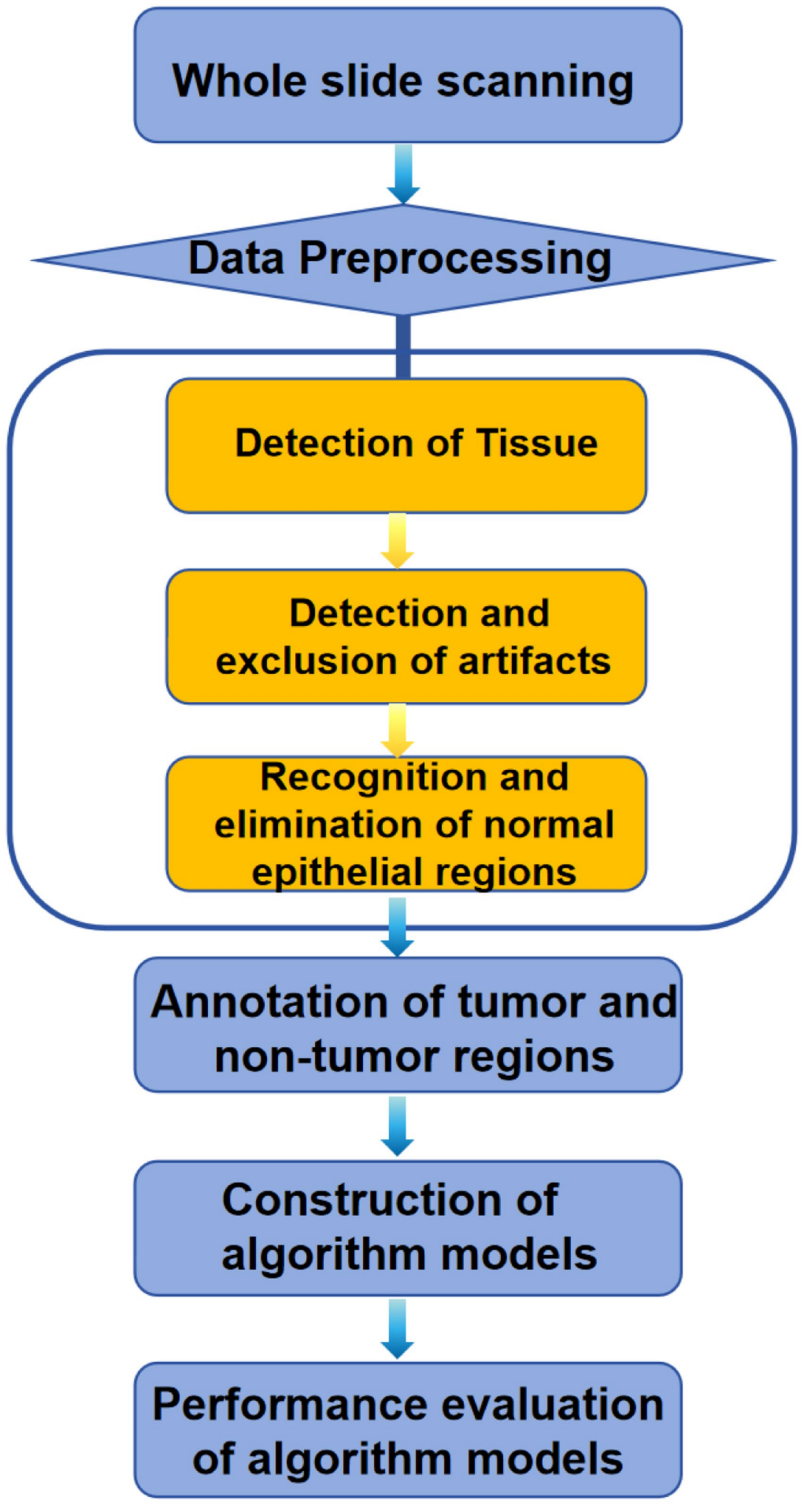
Workflow of the construction CNN models for detecting GSCC in mice.

In this study, forestomach tissue (as described in 2.1) was used to develop CNN models for detecting GSCC in mice, following the workflow below: (1) Whole slide scanning using a digital slide scanner; (2) Automated identification of slide background and tissue regions through Tissue Detection-BF algorithm; (3) Automated identification and exclusion of tissue artifacts through QC Slide algorithm; (4) Recognition and elimination of normal epithelial regions; (5) Annotation of tumor and non-tumor regions in forestomach tissue; (6) Construction of tumor region identification algorithm models; and (7) Performance evaluation of the algorithm models. The workflow is shown in Figure 1.

### 2.4 Data preprocessing

All 149 tissue samples were scanned using a digital slide scanner (NanoZoomer C13210-60, Japan) at a magnification of 40X, and the data was imported into a computer equipped with a 32-core 2.9 GHz Intel Xeon processor 6226R and an Nvidia Quadro RTX6000 graphics card. Data preprocessing was performed using the HALO software on a workstation equipped with an Intel Xeon W-2265 CPU and 64 GB RAM. First, the pre-built algorithm model (Tissue Detection-BF) provided by HALO AI (Version 3.6.4134,) was applied to identify the slide background and tissue regions within the images. Next, the QC Slide algorithm model was used to perform binary classification on the images, identifying artifacts such as bubbles, dust, debris, folds, out-of-focus, and pen-marker, ensuring quality control for all images. The QC Slide and Tissue Detection-BF algorithm models are part of the pre-configured database provided by HALO AI. The QC Slide model, based on the DenseNet architecture, includes a rich training database consisting of hundreds of different types of brightfield slide images, with thousands of annotated artifact regions. Additionally, the training process generates about 2,000 synthetic out-of-focus artifact regions. The QC Slide algorithm performs binary classification on the images, categorizing them into normal tissue and artifact regions. Artifacts include dust/debris, folds, coverslip, out-of-focus, pen-markers, and bubbles. This allows the algorithm to accurately identify normal tissue areas and exclude artifacts, thus enhancing the quality of digital pathology workflows. The Tissue Detection-BF algorithm model, on the other hand, automatically identifies the slide background and tissue areas within images without requiring further training, providing foundational support for subsequent pathological analysis and diagnosis.

Subsequently, the DenseNet AI algorithm model in the HALO AI image analysis software was applied to collect forestomach tissue slide image data containing both normal epithelium and neoplastic epithelium. This ensured that each sample in the dataset had a clear label indicating whether it was normal epithelium. In epithelial tumors, the tissue morphology and cytological features of normal squamous epithelium exhibit certain similarities with tumor tissue, which may lead to unclear boundaries between the tumor and normal epithelium, resulting in false positive or false negative tumor region identification. Therefore, the algorithm model was used for data preprocessing and annotation, ensuring image quality and label accuracy. The annotated regions were then imported into the DenseNet AI algorithm model for algorithm training, with adjustments to parameters such as Cross-Entropy and Iteration during the training process to optimize the model’s performance. Through these processes, the HALO-AI algorithm model was able to recognize regions of normal epithelial tissue in the tissue slides and exclude normal epithelium.

### 2.5 Pathological image annotation

To establish an automated tumor recognition deep learning model, two pathologists manually annotated the tumor regions (primarily refer to areas dominated by squamous epithelial tumor cells) and non-tumor regions (primarily refer to blank areas, stromal regions and necrosis areas) in selected samples of forestomach using the HALO AI Tissue Pathology Image Analysis System (V3.6.4134, Indica Labs, Inc., Albuquerque, NM). Subsequently, the HALO AI DenseNet model was used for training, with the resolution set to 0.5 μm/pixel, and training was halted once the Cross-Entropy converged to 0.1. The model was then used to analyze the entire tissue sections, with the analysis results visually confirmed by pathologist experts. In cases where regions were misidentified by the automated recognition system, these areas were corrected or appropriately annotated. This process enabled the preliminary automatic identification of tumor regions, significantly reducing the burden of image annotation for pathologists.

Next, a new classifier was constructed using the DenseNet AI V2 method, with categories including red (tumor), green (necrosis or stromal), and blue (others, i.e., blank). Once the classification was completed, the tumor tissue was automatically segmented, and the tissue boundaries were annotated accordingly. Based on the classification results, two pathologists (reviewers) manually refined the tumor regions. The entire tissue sections were designated with color-coded numerical labels: red for GSCC, green for necrosis or stromal components, and blue for blank areas.

### 2.6 Construction of tumor region recognition algorithm model

The 149 samples in this study were divided into a training set, validation set, and test set, with images from each set generating 4,944, 827, and 537 patches, respectively (Table 1). The dataset was divided using an 8:1:1 ratio split. A random selection of 119 samples (80%) was assigned to the training set (75 cases of GSCC and 44 cases of normal gastric tissue), 16 samples (10%) were allocated to the test set (10 cases of GSCC and 6 cases of normal gastric tissue), and 16 samples (10%) were assigned to the validation set (10 cases of GSCC and 6 cases of normal gastric tissue). Each algorithm model was trained using the labeled dataset. The validation set was used to evaluate the model’s performance, and model parameters were adjusted to avoid overfitting. The final performance of the model was evaluated on the independent test set.

**Table 1:** Distribution of mouse gastric squamous cell carcinoma data in training, validation, and test sets.

Data	Training set	Validation set	Test set
Tumor patches (individual)	4,825	817	519
Non-tumor patches (individual)	119	10	18
Tumor region area (μm^2^)	2228666624.66	38533264.66	23668576.25
Non-tumor region area (μm^2^)	958664239.31	136273649.37	121293506.69

The FCN, LR-ASPP, DeepLabv3 + and U-Net algorithm models, based on the PyTorch deep learning framework, were trained and validated on the NVIDIA Quadro RTX 6000 GPU. The constructed algorithm model was applied in the test set to identify non-tumor and/or tumor regions in tissue. The FCN architecture is relatively simple, allowing for end-to-end training using a standard convolutional neural network without the need for post-processing steps (17). LR-ASPP incorporates a mirrored ASPP module and a lightweight network structure, enabling performance to be maintained while reducing computational complexity (18). DeepLabv3 + enables multi-scale feature capture and provides good edge detection through its encoder-decoder structure and depth-wise separable convolutions (19). U-Net is a symmetric encoder-decoder structure that uses skip connections to directly transfer high-resolution features from the encoder to the decoder, helping to preserve image details and edge information, making it suitable for small datasets (20). FCN, LR-ASPP, U-Net, and DeepLabV3 + are used with Stochastic Gradient Descent (SGD) as the optimization method. The initial learning rate is set to 0.01 with an adaptive decay of 1e-4. A batch size of 16 is maintained throughout the training process, and the maximum number of epochs is set to 100.

**Figure 2:**
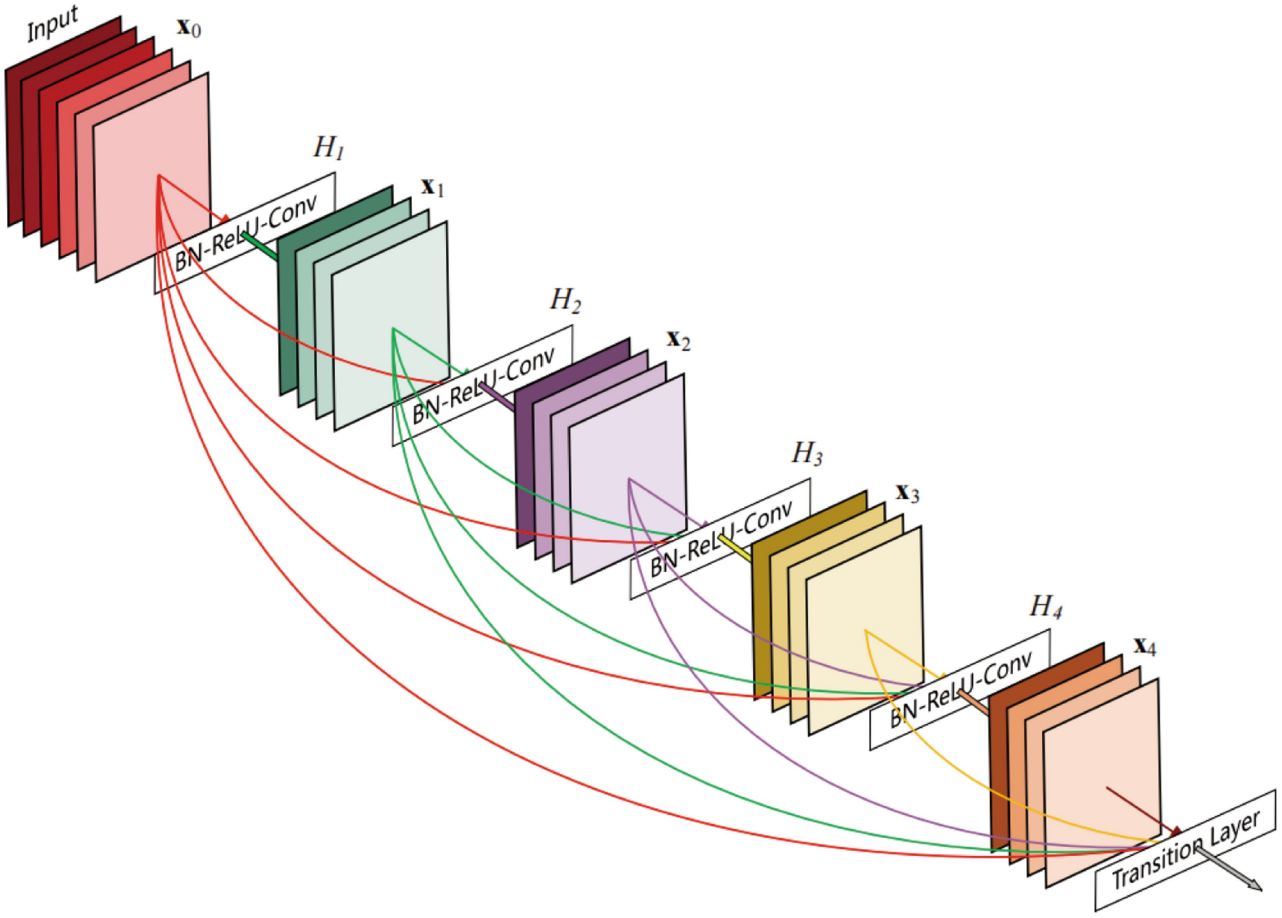
A 5-layer dense block of DenseNet (21).

The DenseNet AI (Plugin) algorithm model was trained and validated using the HALO AI platform and was designed to identify tumor and non-tumor regions in tissue. DenseNet is a densely connected deep neural network structure that maximizes information flow and feature reuse. In DenseNet AI, each layer is connected to the output of all previous layers, allowing the model to better utilize features from earlier layers, thereby improving accuracy. Unlike traditional CNNs, DenseNet enables efficient information flow and sharing across the network through its densely connected architecture, thereby enhancing feature reuse efficiency. Specifically, each convolutional layer receives input feature maps from all preceding layers (not just the immediate previous layer), as illustrated by the dense block connections in Figure 2 (21). This design not only mitigates the vanishing gradient problem but also facilitates feature propagation and optimization, significantly improving model performance on complex tasks. Additionally, DenseNet’s feature reuse mechanism drastically reduces the number of model parameters while boosting training efficiency. Due to this efficient feature utilization, it effectively lowers the risk of overfitting, making it particularly valuable in scenarios with limited data, such as medical image analysis and other data-constrained applications. The DenseNet AI algorithm model in this study was trained at a resolution of 1 μm/pixel, minimum object size of 200 μm^2^ and underwent 46,600 iterations, and training was halted once the Cross-Entropy converged to 0.1.

### 2.7 Performance evaluation of the algorithm models

The performance of the five different algorithm models developed in this study is evaluated using statistical metrics including Precision, Recall, and F1-Score (22, 23). Precision (Pr) refers to the proportion of positive samples among all the samples predicted as positive by the model, also known as the Positive Predictive Value. Recall (Re), also called True Positive Rate, refers to the proportion of samples correctly predicted as positive by the model among all the actual positive samples, reflecting the sensitivity of the algorithm. F1-Score is a valuable evaluation metric because it balances Precision and Recall. The performance of the models is assessed on an independent test set, including metrics such as Precision, Recall, and F1-Score, to validate the generalization ability and practical effectiveness of the models. The Equations 1–3 for calculating the evaluation metrics are as follows:


(1) $$\mathrm{Pr}=\frac{\mathrm{TP}}{\mathrm{TP}+\mathrm{FP}}$$

(2) Re=TPTP+FN

(3) $$F1=\frac{2\times \text{Precision}\times \text{Recall}}{\text{Precision}+\text{Recall}}$$

where True Positive (TP), False Positive (FP), True Negative (TN), and False Negative (FN) represent cases when the model predicts the positive class as positive (i.e., TP) or as negative (i.e., FN) and predicts the negative class as positive (i.e., FP) or as negative (i.e., TN), respectively.

## 3 Results

### 3.1 Pathologists’ diagnosis for GSCC

**Figure 3:**
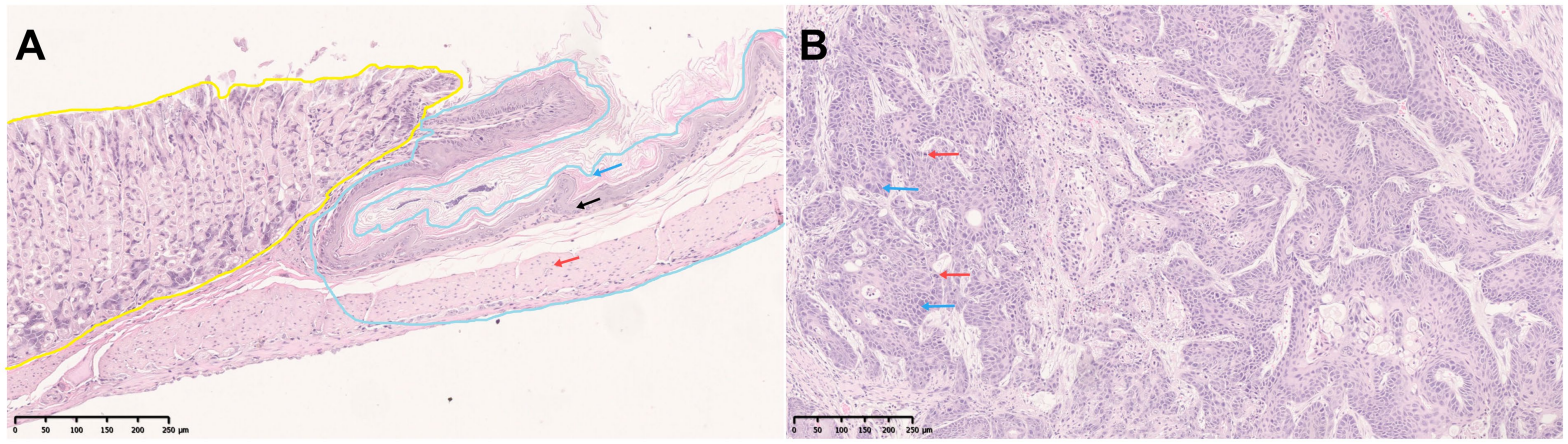
Gastric tissue of C3H mice. **(A)** The normal gastric tissue of mice, including the forestomach (blue) and glandular stomach (yellow) (black arrow: basal layer; red arrow: muscular layer; blue arrow: keratinized layer). **(B)** Mouse gastric squamous cell carcinoma (blue arrow: atypical cells; red arrow: mitotic figures).

Two pathologists performed diagnoses on the digital scanned slides of gastric tissue from 149 mice independently and the results showed diagnostic consistency/consensus. Based on the diagnostic criteria for GSCC, 56 cases of normal mouse gastric tissue and 93 cases of GSCC were confirmed. The normal gastric tissue of the mice was categorized into the forestomach and glandular stomach. The normal forestomach is covered by squamous epithelium, consisting of stratified squamous epithelium, where the cellular structures of the basal layer, muscular layer, and keratinized layer are clear and normal (Figure 3A). Cases diagnosed as GSCC exhibit malignant tumor characteristics, including individual tumor cells or small clusters of tumor cells breaching the basement membrane and displaying invasive growth. There is a loss of cellular differentiation with pronounced atypia, hyperchromatic nuclei, increased cell volume, and distinct nucleoli. Mitotic figures are also observed (Figure 3B).

### 3.2 Preprocessing results of pathological image data

**Figure 4:**
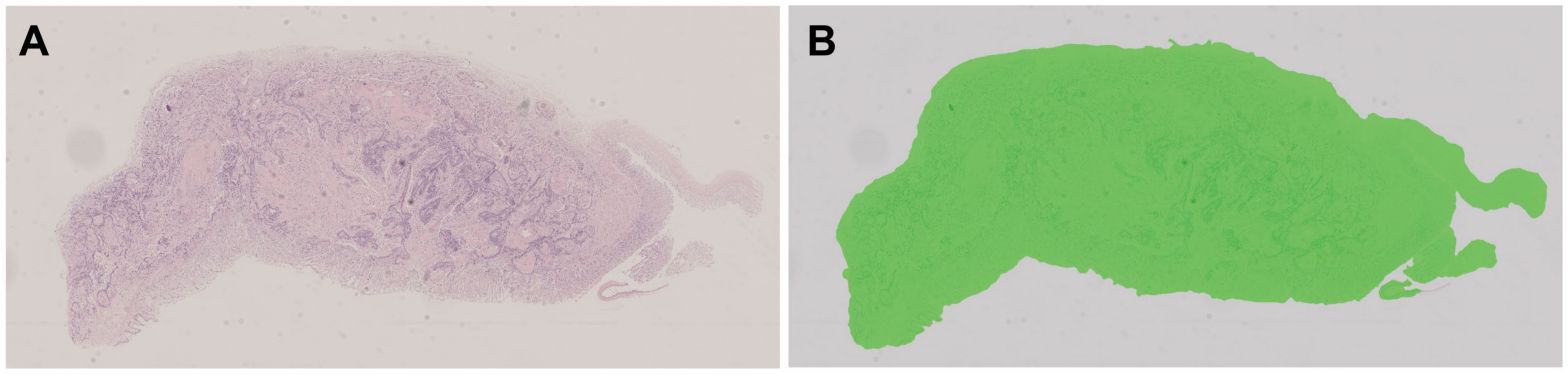
The background and tissue regions of slides were identified based on the HALO AI Tissue Detection-BF algorithm model. **(A)** The original pathological data image; **(B)** The result of the recognition based on Tissue Detection BF algorithm model, with the tissue area being green and the slide background being gray.

**Figure 5:**
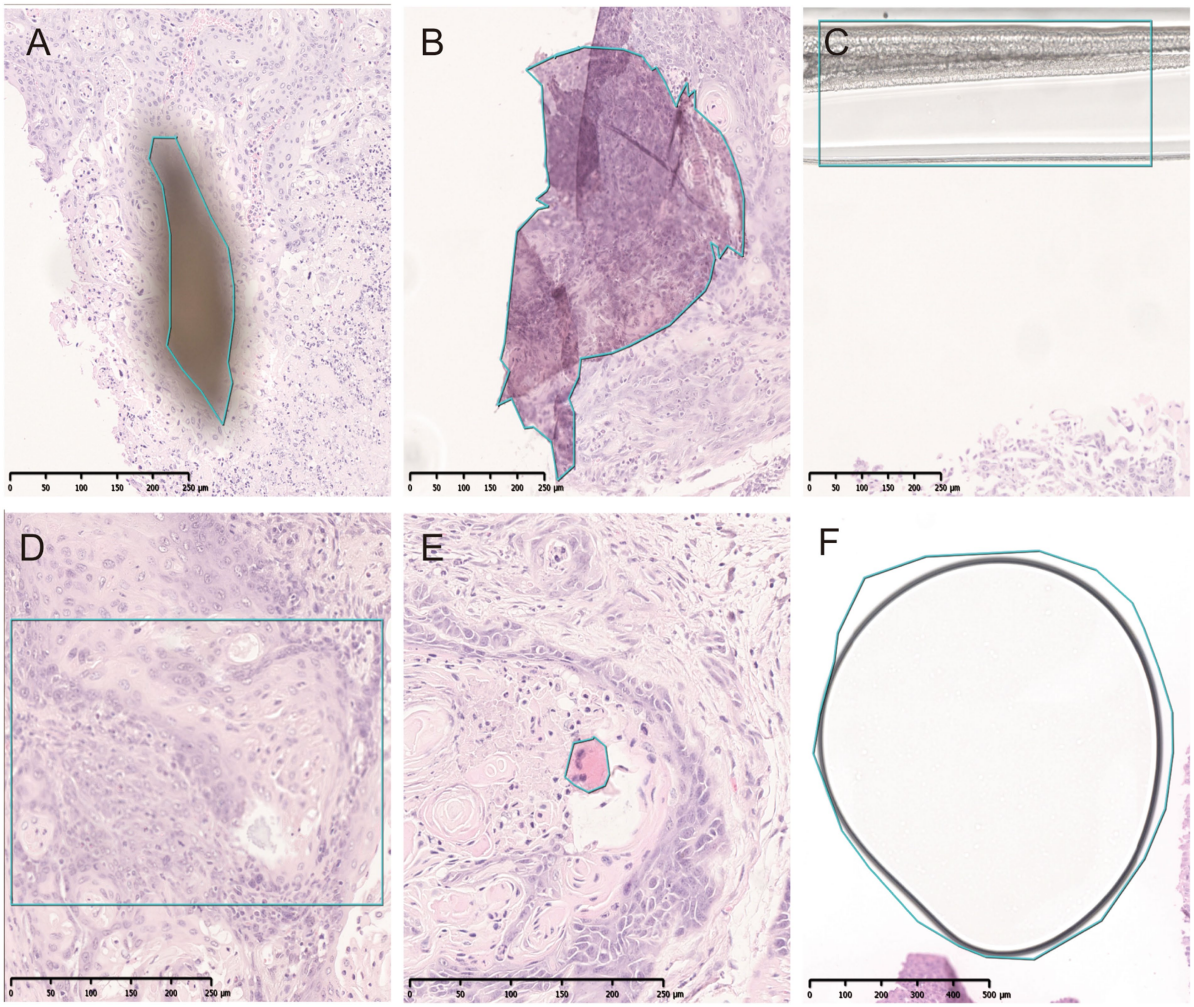
Artifacts identified by QC slide. **(A)** Dust. **(B)** Folds. **(C)** Coverslip. **(D)** Out-of-focus. **(E)** Pen marker. **(F)** Bubble.

**Figure 6:**
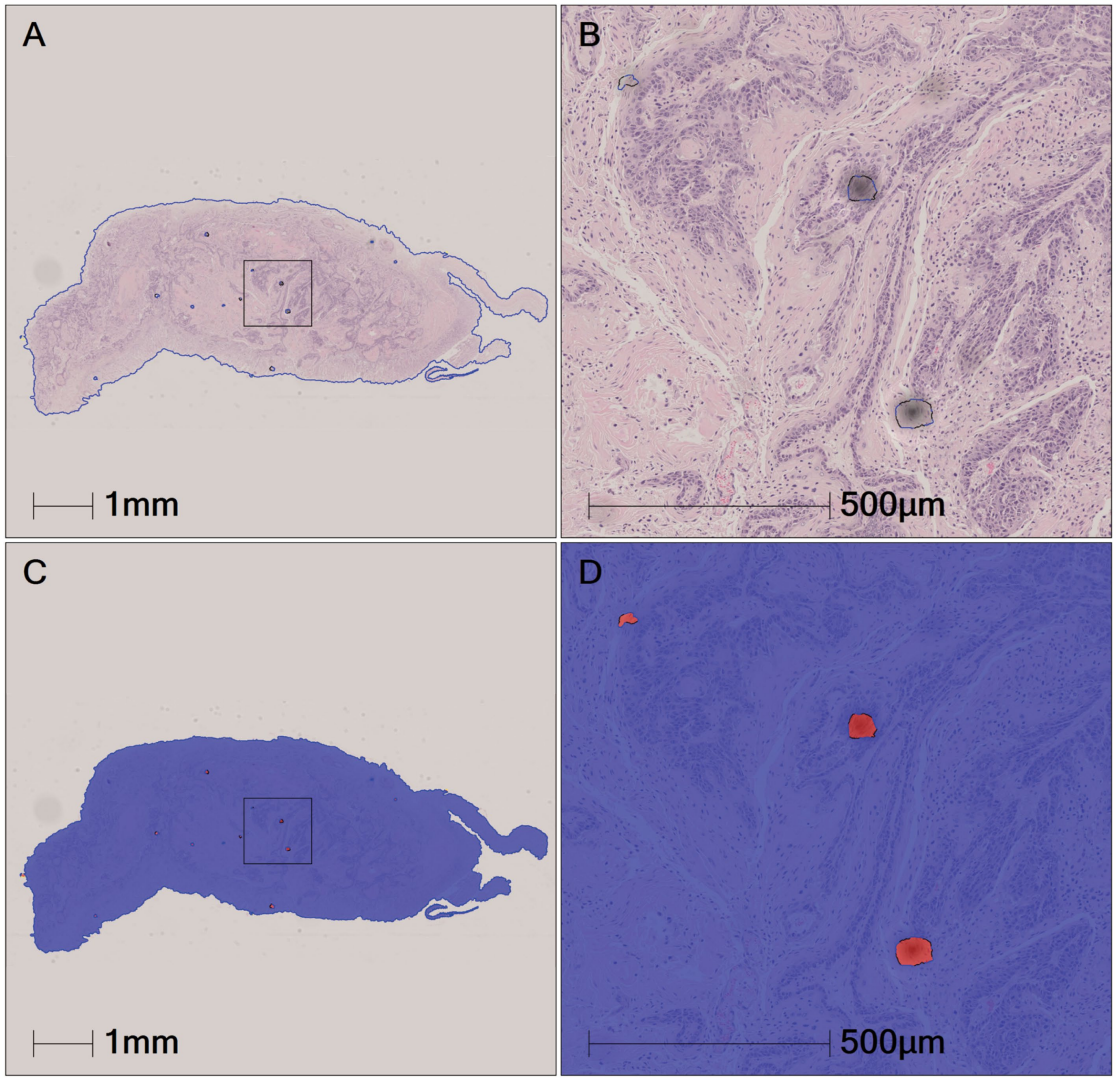
The artifact regions were identified in tissues based on the HALO AI QC slide algorithm model. **(A)** The original pathological data images and dusts. **(B)** High-magnification micrograph of dusts. **(C,D)** The artifacts identified by HALO AI, with red areas indicating dust artifact.

All pathological image data was processed using the algorithm model (Tissue Detection-BF) from the pre-configured database provided by HALO AI, which automatically identifies the slide background and tissue regions in the images (Figure 4), with the green markings indicating the results recognized by the BF algorithm. Subsequently, the QC Slide algorithm model provided by HALO AI was applied to eliminate artifacts within the tissue regions (Figure 5), and we took dust as an example to demonstrate how QC Slide identified artifacts (Figure 6).

**Figure 7:**
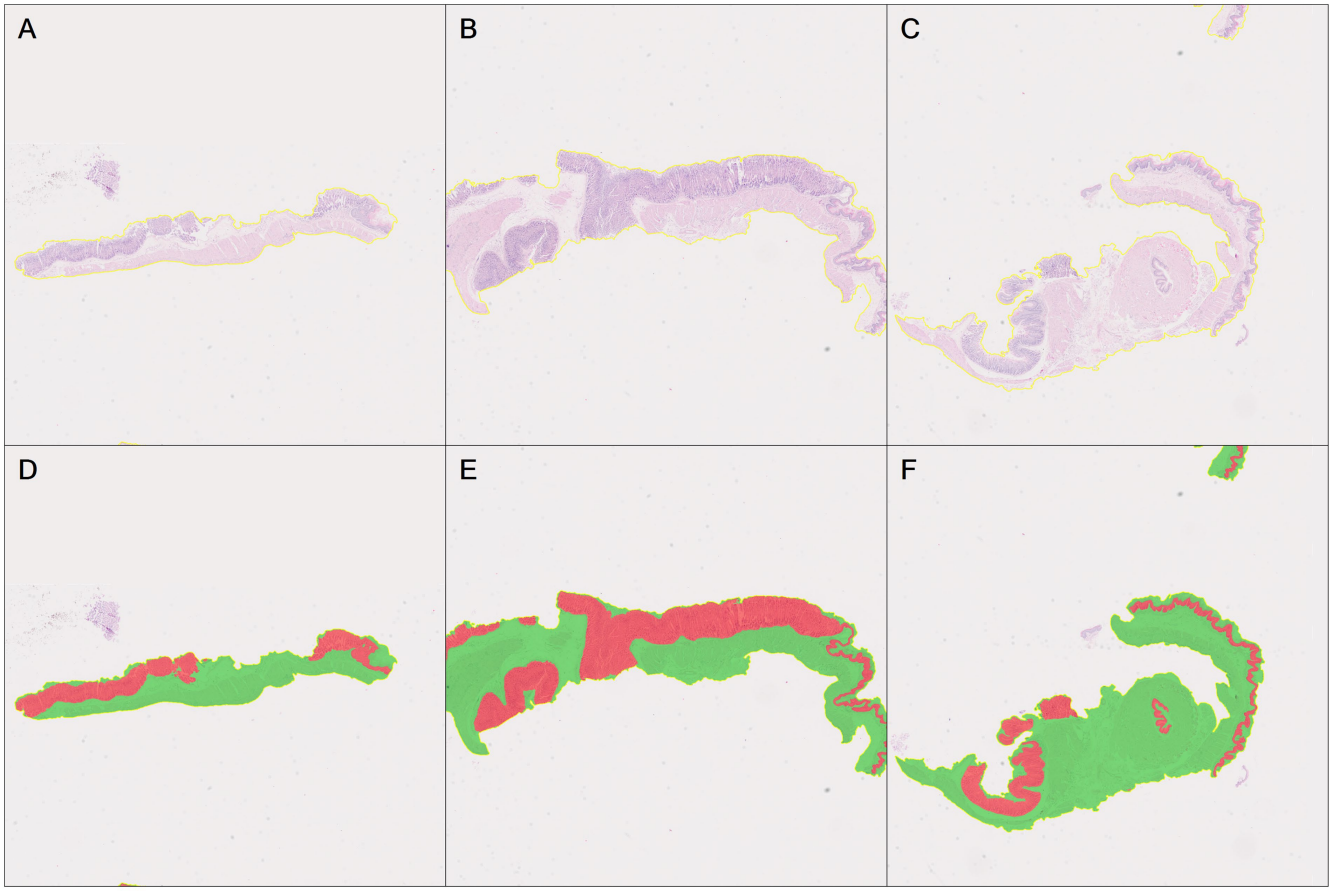
Automatic recognition of the normal epithelial tissue and other tissue based on DenseNet AI of HALO AI. **(A–C)** The original pathological images of three normal mouse gastric tissues. **(D–F)** Correspond to HALO automatically annotating images of three normal gastric tissues **(A–C)**, respectively. Red represents the normal epithelial tissue automatically recognized by HALO, while green represents normal other tissues.

Subsequently, based on the DenseNet AI of HALO AI algorithm model, normal epithelial tissue regions were identified, correctly annotated, and preprocessed, removing regions automatically recognized as normal epithelium (marked in red) (Figure 7). The annotated regions were then included in the HALO AI DenseNet model for algorithm training.

### 3.3 Annotation of digital images by pathologists

**Figure 8:**
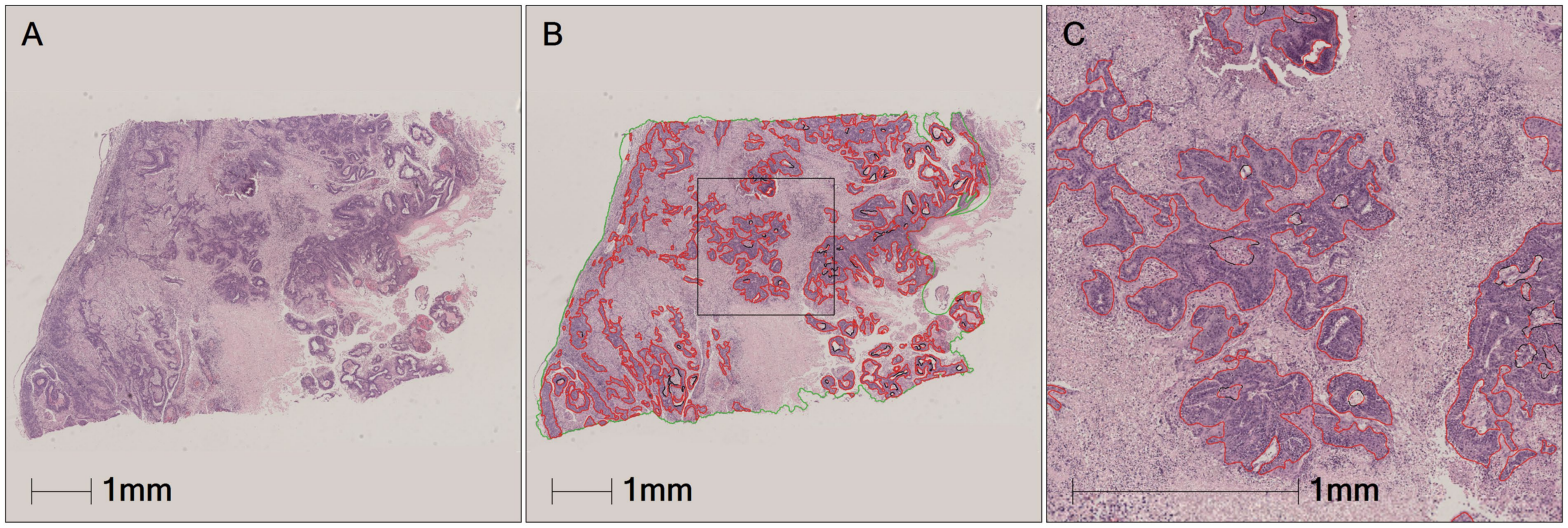
Tumors area manually annotated by pathologist. **(A)** The original pathological data image. **(B)** Green is tumor area and red is the tumor area automatically annotated by HALO AI. **(C)** Black is the tumor area manually revised and annotated by pathologist.

Two pathologists manually refined the tumor annotations on all positive cases (93 GSCC images) based on semi-automatically annotated tumor regions by the annotators, creating accurate ground truth data, i.e., pathologist-revised labels (Figure 8). The annotated pathological image data was subsequently used for the construction of the algorithm models.

### 3.4 Validation and testing results of the algorithm models

To investigate the optimal deep learning algorithm for assisting the diagnosis of GSCC in mice during carcinogenicity studies, we compared five different algorithms based on different architectures: FCN, LR-ASSP, DeepLabv3+, U-Net, and DenseNet, for detecting GSCC. The above algorithms were trained on the training set, and their performances were ultimately evaluated on the independent test set.

**Figure 9:**
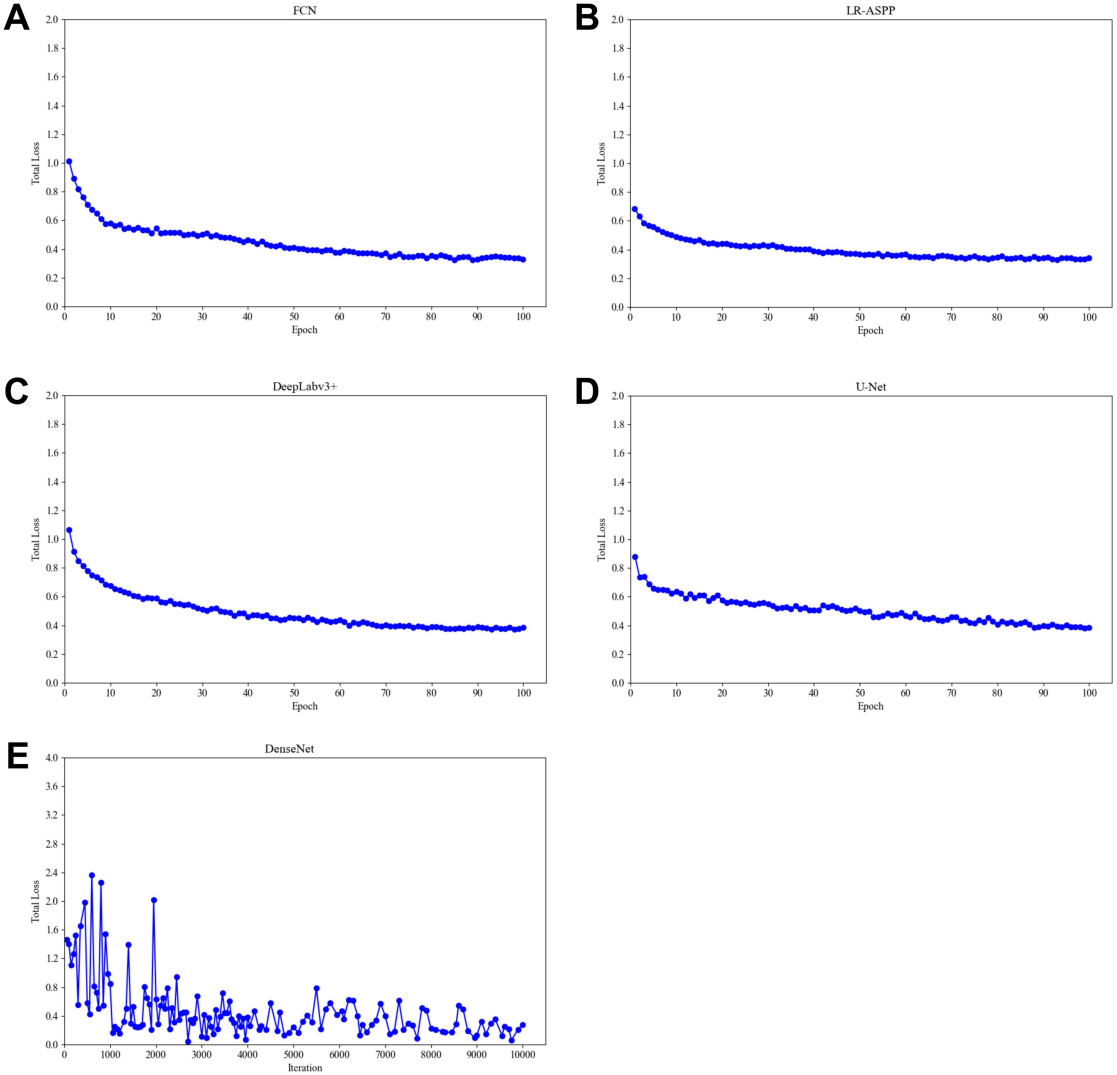
Total training loss during training of different algorithm models. **(A)** The total loss function diagram of the FCN model. **(B)** The total loss function diagram of the LR-ASSP model. **(C)** The total loss function diagram of the DeepLabv3 + model. **(D)** The total loss function diagram of the U-Net model. **(E)** The total loss function diagram of the DenseNet model.

**Figure 10:**
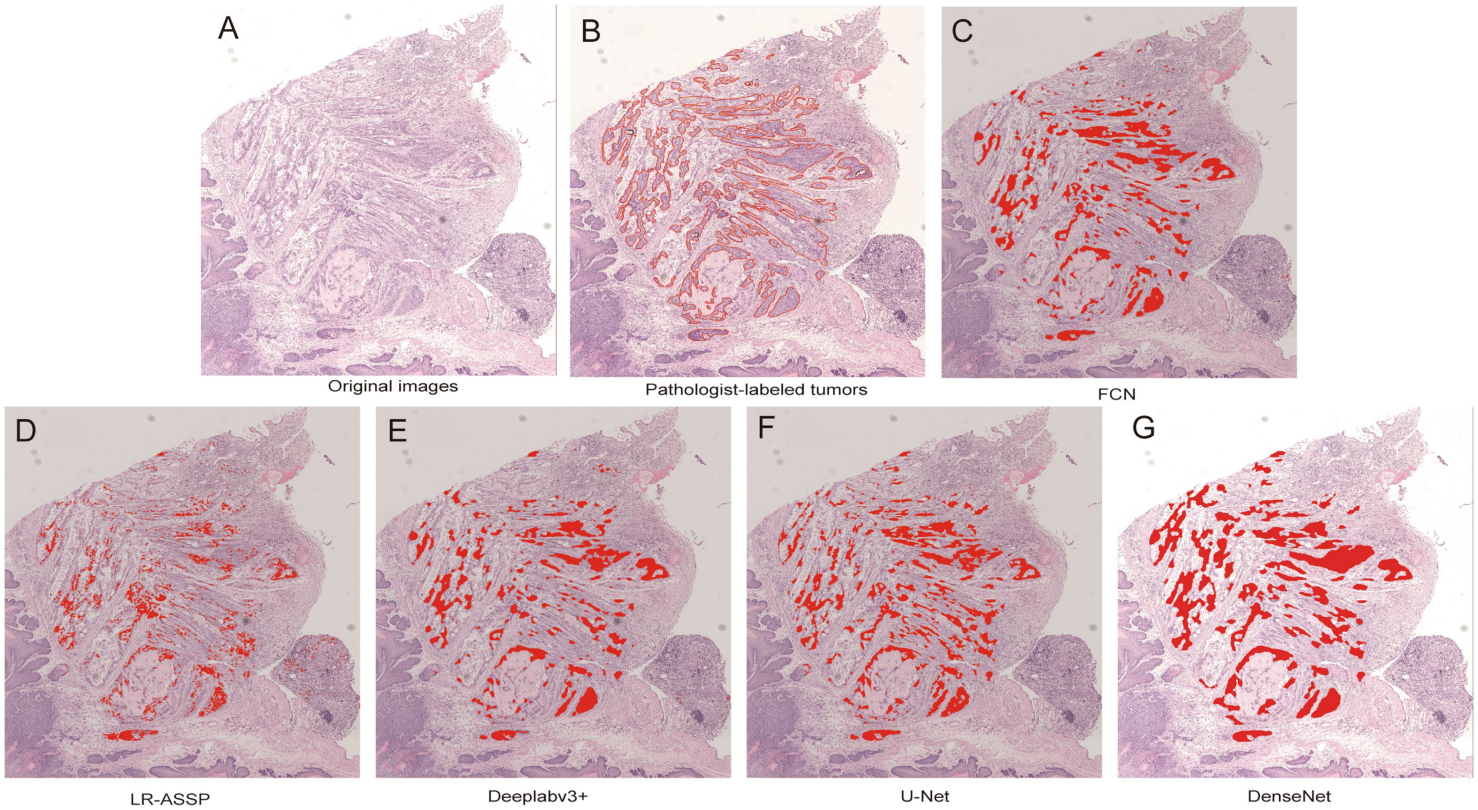
Comparison of original, annotated, and algorithm predicted lesion results based on a GSCC image. In the picture, the red line marked by the pathologist outlines the area of gastric squamous cell carcinoma. **(A)** Original images. **(B)** Pathologist-labeled tumors. **(C–G)** The gastric squamous cell carcinoma predicted by FCN, LR-ASSP, DeepLabv3+, U-Net, and DenseNet was marked in red, respectively.

During the training process, the total loss value of each model was monitored. Although the loss components calculated by different algorithms varied during training, the loss values for all five algorithms rapidly stabilized in the early stages of learning (Figure 9). As a result, each algorithm was successfully trained using the training dataset. The trained algorithm models effectively delineated the tumor regions of GSCC (Figure 10).

**Figure 11:**
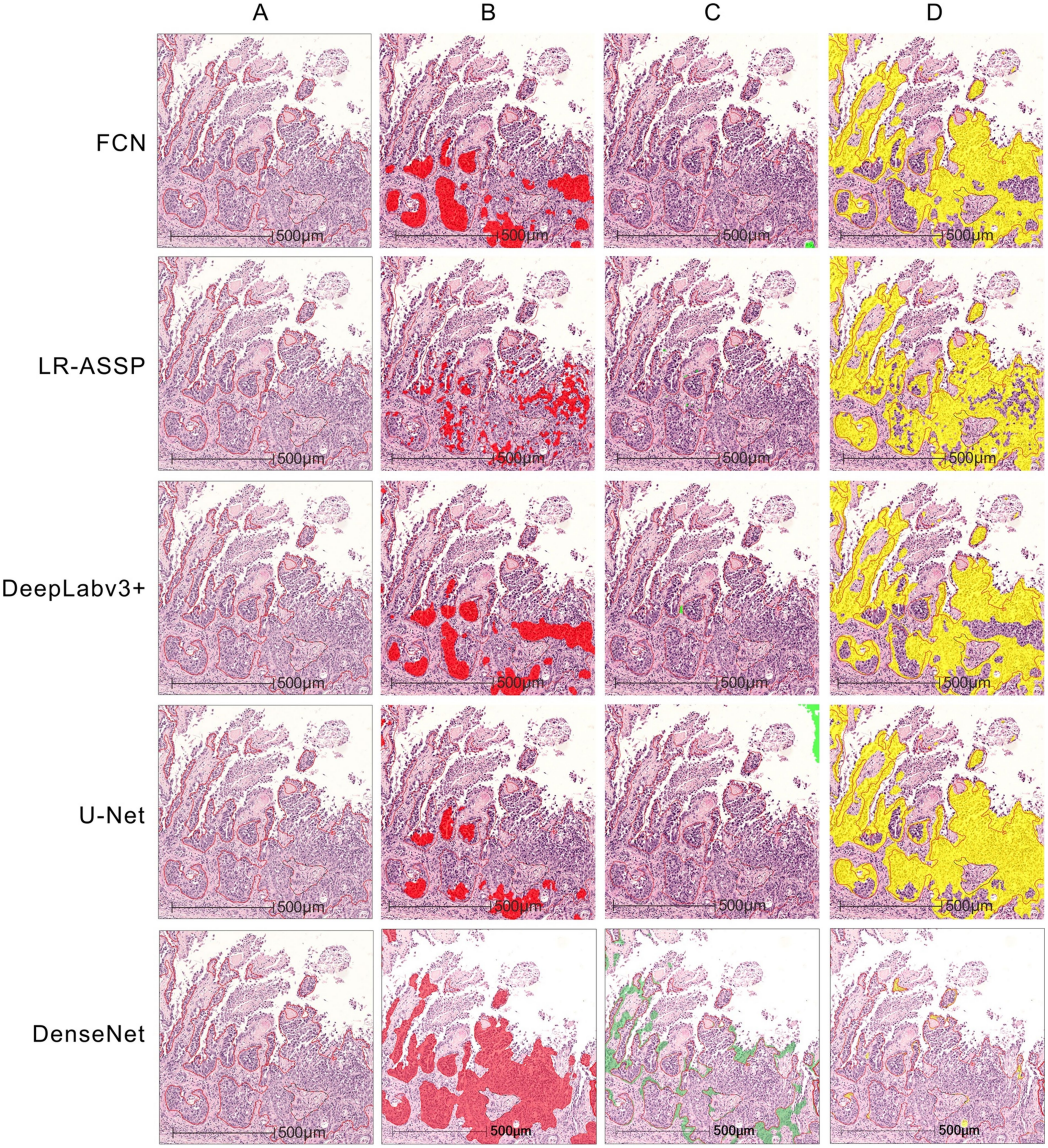
The example of test results for the DenseNet AI algorithm model. **(A)** The tumor area manually annotated by pathologists. **(B)** The tumor region predicted by the algorithm models—tumor true positive. **(C)** The tumor region predicted by the algorithm models—tumor false positive. **(D)** The non-tumor region predicted by the algorithm models-tumor false negative.

The performances of the five constructed algorithm models were evaluated on the test set as Precision, Recall and F1-Score (Table 2). On the test set, the DeepLabv3+, U-Net, and DenseNet models demonstrated higher overall performance, with F1-scores ≥90%. As shown in the Figure 11, the tumor regions predicted by these three models closely resembled the ground truth annotations, outperforming the predictions of the FCN and LR-ASPP models.

**Table 2:** Performance results of the five algorithm models.

Algorithm models	Precision (final model)	Recall (final model)	F1-score (final model)
FCN	0.8805	0.8749	0.8777
LR-ASSP	0.807	0.7954	0.8012
DeepLabv3+	0.9027	0.9032	0.9029
U-Net	0.9347	0.8934	0.9136
DenseNet	0.9044	0.9291	0.9157

Precision indicates the proportion of correct predictions made by the model compared to the actual results. Recall reflects how closely the model’s predictions align with the actual cases of GSCC. In medical diagnostics, recall is particularly crucial as it indicates the model’s ability to identify true positives. The results of this study revealed that U-Net had a slightly lower Recall rate compared to DenseNet. However, despite the model’s strong predictive performance for tumors, there were instances where non-tumor regions were misclassified as tumor regions (Figure 11C) and tumor regions were misclassified as non-tumor regions (Figure 11D). The DenseNet model predicted the highest number of true positives (red regions) and the fewest false negatives (yellow regions), consistent with its highest recall rate (Figures 11B,D). Other models misclassified tumors within necrotic or stromal components as non-tumor regions, resulting in higher rates of false negatives.

## 4 Discussion

It has been reported that anthracycline-based antitumor drugs, antiarrhythmic drugs, non-steroidal anti-inflammatory drugs, and herbs such as Aristolochia can induce gastric cancer (24–26). According to the requirements of the *International Council for Harmonization of Technical Requirements for Pharmaceuticals for Human Use* (ICH) S1B (R1) guideline and OECD guideline for the testing of chemicals: carcinogenicity studies, carcinogenicity studies are performed to assess the carcinogenicity potential of numerous compounds such as pharmaceuticals and chemicals (27, 28). Rodents, particularly mice, are commonly used for carcinogenicity studies. GSCC can occur as spontaneous and drug-induced change in the forestomach of mice, and toxicologic pathologists primarily rely on microscopy to perform pathological diagnoses for hundreds of animals in the context of carcinogenicity studies to evaluate the carcinogenic potential of the tested compound. However, for a century, the working mode of pathologists has seen little advancement, as manual slide reading, a qualitative analysis method, is highly subjective. The accuracy of lesion diagnosis is greatly influenced by factors such as pathologists’ qualifications, educational background, work experience, and work conditions (29). Additionally, the large volume of pathological slide reading in rodent carcinogenicity studies and the tightness of drug development timeliness can lead to diagnostic drift. Therefore, the use of deep learning CNN algorithms for auxiliary diagnosis of GSCC in mice is of significant importance to improve the consistency and accuracy of toxicological pathology diagnoses (30, 31).

In recent years, AI technologies have made continuous breakthroughs in fields such as image recognition, dermatological lesion identification, and pathological slide analysis. The advent of deep learning has particularly overcome the limitations of manual feature extraction, which is often inefficient and incomplete. Deep learning has become the go-to method for medical image analysis, primarily employed in tasks such as image classification, object detection, segmentation, registration, and other related applications. It has been widely adopted across diverse medical domains, including neurological imaging, retinal scans, pulmonary diagnostics, digital pathology, breast imaging, cardiac studies, abdominal examinations, and musculoskeletal assessments (32). CNN, one of the representative architectures of deep learning, are inspired by the structure of the brain’s visual cortex and visual activity principles. CNNs consist of multiple image perceptrons, several neural network layers, continuous convolution layers, and pooling layers, which enable deep learning from raw image data to predict feature classifications and thus recognize medical images (33). CNN-based models are often seen as “black-box” models in many tasks, including semantic segmentation, meaning their decision processes are hard to understand. Explainability and visualization techniques have become important tools to solve the black-box nature of these models, i.e., Explainable Artificial Intelligence (XAI), including Feature Visualization, Class Activation Mapping, Saliency Mapping, Prediction Difference Analysis, Grad-CAM, Trainable Attention, Guided Grad-CAM, Deconvolution, Meaningful Perturbation, SHAP, Attention and so on (34). CNN is progressively transforming histopathology. Researchers have developed evaluation frameworks to assess the robustness of CNN-based cancer classification models against staining variations by testing them on WSI of breast, gastric, and colon cancers (35). AI and machine learning can reduce or eliminate pathologists’ error or inconsistency rate in describing microscopic histopathological features. Some models already match pathologists in WSI diagnosis. For example, AI-assisted pathologists achieve 99.5% accuracy in identifying metastatic breast cancer cells in lymph nodes, surpassing individual human (96%) or AI-only (92%) diagnoses (36). A study using CNNs for melanoma detection showed higher sensitivity, specificity, and accuracy than 11 pathologists (37). Some previous studies have reported that in the detection or diagnosis of diseases such as colorectal adenoma, breast cancer, lung adenocarcinoma, gastric cancer, etc., the diagnostic or predictive results based on AI models may outperform or be comparable to the diagnoses made by pathologists, as shown in Table 3. Although some AI models now rival the diagnostic performance of pathologists, their application remains dependent on pathologists’ annotations and definitions of histopathological alterations. Therefore, the collaborative integration of pathologists’ advanced cognitive expertise with AI’s ability to perform repetitive tasks rapidly and accurately is expected to drive transformative advancements in toxicologic pathology. Consequently, integrating AI into pathology workflows does not replace pathologists but serves to enhance their efficiency and diagnostic accuracy. Recently, Scholars around the world have applied deep learning CNN models to classify gastric cancer (overall types), gastric adenocarcinoma, and precancerous lesions, demonstrating high sensitivity and specificity. These models can serve as an auxiliary diagnostic and screening system for clinical gastric biopsy specimens (38, 39). However, the type of gastric cancer in mice differs from the common gastric adenocarcinoma found in humans. Because the forestomach (a rodent-specific region) is lined by squamous epithelium, it is predisposed to developing squamous cell carcinoma. Currently, there have been no reports on the use of deep learning CNN models for the auxiliary diagnosis of gastric cancer in mice.

**Table 3:** AI algorithm models vs. pathologists: performance comparison in image diagnosis.

Reference	Image types	Purpose	Algorithm	Results summary
Song et al. (41)	WSI	Detect colorectal adenoma	CNN (DeepLab v2; ResNet-34)	The best DL model reached an AUC of 0.92, showing comparable performance to the pathologists, even better than the average pathologist
Lin et al. (42)	H&E stained WSI	Detect lymph node metastases of breast cancer	Modified FCN (Fast ScanNet)	The performance of the FCN model superpassed the pathologists (FROC 0.8533 vs. 0.7325; AUC 0.9935 vs. 0.9660)
Wei et al. (43)	WSI	Classify of histologic patterns in lung adenocarcinoma	CNN (ResNet)	The CNN model slightly outperformed pathologists (average kappa score:0.525 vs. 0.485; average agreement:66.6% vs. 62.7%)
Jeong et al. (44)	H&E stained WSI	Predict Epstein–Barr virus associated gastric cancer	EBVNet (ResNet50 and Inception v3)	The DL model demonstrated superior diagnostic performance compared to pathologists (AUROC 0.88 vs. 0.75; AUPRC 0.65 vs. 0.41)
Kim et al. (45)	ICH stained WSI	PD-L1 CPS scoring	Aperio IHC image	AI model was 84.6% concordance with pathologists

WSI, whole slide image; CNN, convolutional neural network; DL, deep learning; AUC, area under the curve; FROC, free-response receiver operating characteristic; AUROC, area under receiver operating characteristic curve; AUPRC, area under recall precision curve; ICH, immunohistochemistry; PD-L1, programmed death-ligand 1.

This study applied five commonly used CNN models to identify mouse GSCC in a non-clinical research environment. Algorithm models were established through training and validation on multiple gastric tissue samples and evaluated by comparing the results with pathologist-annotated slide images. Our findings indicate that the DenseNet model achieved the highest Recall rate and F1-score, demonstrating superior performance. This confirms that the DenseNet model exhibits strong generalization capabilities and practical efficacy in predicting both tumor and non-tumor regions in mouse GSCC. Before constructing the tumor recognition algorithm model, we considered factors such as background interference, tissue artifacts, and normal normal epithelial tissue, which could affect the accurate identification of tumor regions. To improve the accuracy and efficiency of tumor identification/classification, we developed an automated tumor recognition strategy. This strategy utilizes the HALO AI-based Tissue Detection-BF algorithm, Slide QC algorithm, and DenseNet algorithm to identify slide background and tissue regions, eliminate artifacts and benign tissues, and optimize parameters after data preprocessing, achieving precise localization and annotation of the GSCC regions in pathological tissue images.

In our test set, the Precision, Recall, and F1-scores of the DeepLabv3+, U-Net, and DenseNet AI models were all above 90%, demonstrating good tumor region recognition performance similar to the true images annotated by pathologists. The U-Net model, originally designed for medical imaging, exhibited the highest precision in this study, demonstrating excellent tumor prediction ability, but its Recall rate was lower than that of the DenseNet model. Recall rate refers to the true positive rate, which is the proportion of positive samples predicted by the model compared to actual positive samples. A higher true positive rate indicates better performance in identifying positives, which is crucial for pathological diagnostic support. The DenseNet algorithm model achieved the highest recall rate, reflecting its ability to predict fewer false negatives compared to other models. In other words, the DenseNet model helped correctly diagnose more positive samples as tumors, despite some false positives remained. Overall, this improved detection sensitivity. DenseNet outperforms FCN, U-Net, and DeepLabV3 + in terms of performance, primarily due to its unique dense connection structure. Compared to FCN, DenseNet improves model accuracy and stability by enhancing feature reuse and gradient propagation. Compared to U-Net, DenseNet’s dense connections facilitate smoother information flow, more efficient feature reuse, and prevent information loss, thus improving accuracy. Compared to DeepLabV3+, DenseNet’s stronger feature reuse and gradient propagation capabilities enable better generalization on tumor datasets, resulting in higher accuracy.

The DenseNet network model, proposed by Huang et al. (21), is a CNN that combines the advantages of ResNet and GoogLeNet algorithms. The main feature of DenseNet is its ability to address the vanishing gradient problem in deep CNN. It ensures that each feature layer in every Dense block is fully connected, allowing the input of each feature layer to be linked with the output of all previous layers. This fully represents both shallow and deep features, effectively mitigating gradient vanishing caused by deeper layers and enhancing the model’s resistance to overfitting. In this study, the DenseNet network model underwent 46,600 iterations, with a Cross-Entropy parameter of 0.397. The final F1-score of the model was 0.916. In the test set, the model still showed some false positives and false negatives. This may be due to the strong global characteristics of tumor regions, with malignant cells being either clustered or scattered. Well-differentiated tumor cells resemble normal epithelial cells in texture and color, and the lesions have irregular morphology with more detailed features. As a result, necrosis near the tumor region in some images was misclassified as tumor tissue, leading to an increase in false positives and lowering the precision of tumor region recognition.

## 5 Conclusion

This study represents an initial exploration of applying artificial intelligence technology in the auxiliary diagnosis of mouse GSCC. The DenseNet algorithm model established in this research can effectively identify tumor and non-tumor regions in mouse GSCC pathology images. The model’s performance evaluation also yielded favorable results, but there are still limitations, especially in terms of precise tumor region recognition, which requires further exploration. In subsequent research, we plan to increase the number of cases, expand the sample pool from multiple institutions, and improve the model’s generalization ability. Additionally, Research study will enhance data quality to ensure that pathology images with excellent slide preparation, staining, and accurate annotations are included in the training set, as high-quality data determines the upper limit of model performance (40). Furthermore, this study has focused solely on the determination of malignant tumors. In future research, we will incorporate atypical hyperplasia pathology images and images of squamous cell carcinoma with varying degrees of differentiation will broaden the model’s applicability. Finally, the model’s performance needs further exploration and optimization to truly reach the diagnostic level of pathologists. Overall, the application of AI technology in non-clinical safety evaluation can assist toxicological pathologists in making rapid diagnoses, improving efficiency, precision, and consistency, reducing subjectivity in diagnoses, and supporting drug development.

## Data Availability

The original contributions presented in the study are included in the article/supplementary material, further inquiries can be directed to the corresponding authors.
